# Complete Suppression of Urine for Seven Weeks

**Published:** 1831-04-01

**Authors:** 


### COMPLETE SUPPRESSION OF URINE FOR SEVEN WEEKS.

WE notice this case, as published in a late number of Hufeland's journal, for reasons which will appear in the sequel.

*Case.* Itzig, a boy, 12 years of age, and who had always enjoyed good health, was taken, in the latter days of

December with a kind of malaise, which lasted five or six days, and for which he took some purgative medicine, which acted also as an emetic. From this date the urinary secretion ceased entirely, not one drop of urine being passed, although the boy drank freely of liquids. Once only he passed about a pint of urine, after taking some diuretic medicines—and then the secretion stopped. Dr. Hubenthal was then consulted, and prescribed various medicines during a space of nineteen days, without any success. At the end of seven weeks, viz. on the 20th of Feb. Dr. Ramm visited the patient, and found that no urine had passed for many weeks — that a natural motion of the bowels took place every second or third day—that there was no increase of perspiration, but the reverse—that the appetite was tolerable, and sleep sound. The boy in all other respects appeared healthy. There was no reason to suspect any deception on the part of the boy or his parents. On the 22d Feb. there was a consultation of several physicians. The abdomen was rather distended, as there had been no stool for some days. A catheter was introduced into the bladder, but not a drop of water flowed. Injections of castor oil with oil of turpentine, were thrown up per anum, and turpentine frictions were applied over the loins, and bals. copaibæ was ordered internally. These remedies were commenced on the evening of the 22d, and on the 24th, a few drops of urine mingled with blood were passed. In a few hours more a quart of water was discharged, and this was repeated every day afterwards.

Most people will be sceptical as to the truth of the above narrative; and we should have doubted its authenticity ourselves, had we not lately seen a case where some weeks passed without any urine being secreted, and where it was impossible that any deceit could be practised. The catheter was introduced almost every day, but nothing except a few drops of blood followed. There was no affection of the head all this time; but a tumour existed in the right side of the abdomen, very painful, and apparently of a malignant nature. We this day (6th Jan. 1831) examined the patient, and ascertained that not a drop of urine had passed for five weeks! Mr. Acret can attest the truth of this assertion, strange as it may appear.*

* Since the above was written, the tumour burst internally—a large quantity of fluid was discharged per anum—and the patient is recovering.—*Ed.*

